# Editorial: Meditative practice and behavioral neuroscience

**DOI:** 10.3389/fnbeh.2023.1304210

**Published:** 2023-11-24

**Authors:** Junling Gao, Chunqi Chang, Liye Zou, Eric W. Tsang

**Affiliations:** ^1^Buddhist Practices and Counselling Science Lab, Centre of Buddhist Studies, The University of Hong Kong, Hong Kong, China; ^2^School of Biomedical Engineering, Shenzhen University, Shenzhen, China; ^3^Department of Psychology, Shenzhen University, Shenzhen, China; ^4^Faculty of Education, The University of Hong Kong, Hong Kong, China

**Keywords:** mindfulness meditation, machine learning, awareness training program, wearable EEG, brain-heart connection, mind body coherence

Mental wellbeing is paramount in today's global society, emphasized by advancements in brain research and algorithms. Major global players, including the US, EU, and China, are heavily investing in this domain. Yet, understanding concepts like consciousness and the mind-body problem remains elusive. Disorders like depression and epilepsy still challenge us. Ancient practices might offer insights into these modern dilemmas. This issue delves into the mind-body connection through a neuroscientific lens.

The elusive nature of mind sometimes clashes with scientific objectivity, necessitating a balance between subjectivity and objectivity. Recognizing the intrinsic mind-body connection, which has been present since life's inception, is crucial. This calls for a holistic research approach, and meditation, an age-old mental training practice, might be the key. This issue emphasizes meditative practices within the realm of behavioral neuroscience. Meditation, spanning various forms like Yoga, religious chanting, and contemporary mindfulness, has been practiced for millennia. Intensive training in meditation by practitioners, especially Buddhists and Hindus, can lead to profound mental states like enlightenment. While reaching such states is challenging, meditation's benefits, such as improved mental clarity and problem-solving, are well-documented. The rising popularity of mindfulness meditation, supported by scientific validation, underscores the need to further decipher its mechanisms.

A practical approach involves examining meditation from a brain-heart perspective. This dual training, linking brain and cardiac activities, might be key, especially in today's world where mental and physical aspects are often segregated. For instance, office workers might focus mentally, neglecting their body's needs. Persistent mind-body separation can be detrimental. Meditation can bridge this gap, enhancing overall wellbeing. Several studies in this issue found increased mind-body interaction post-meditation (Gan et al.; Gao, Sun et al.; Wong et al.), suggesting that studying the brain-heart connection can provide insights into the mind-body problem.

This issue encompasses diverse research areas, from epilepsy to mindfulness, utilizing various neuroimaging tools like EEG, fMRI, and fNIRS. The goal is to probe brain function using both objective tools and subjective mind states, especially during meditation. In this Research Topic, the emphasis is on mental wellbeing and brain-heart connection, which are related to mind-body problem in a broader sense. Its intricate relationship with meditation is evident. Given the prominent proportion of mindfulness-related research, a significant portion of the research, such as the studies on “*Enhanced resting-state functional connectivity*” (Gan et al.) and “Enhancing Chinese preschoolers' executive function via mindfulness training” (Xie et al.), delves deep into the realm of mindfulness application. These studies, along with others on “Interoceptive awareness” (Guu et al.) and “Increased neurocardiological interplay after mindfulness meditation” (Gao, Sun et al.), investigate mindfulness from varied yet interconnected angles, highlighting its central role in meditation-related neuroscientific exploration.

However, the scope of this issue isn't limited to mindfulness alone. Traditional meditation techniques, deeply rooted in Buddhist theories, also find their place. The “Loving-kindness meditation (LKM) modulates brain-heart connection” (Wong et al.) is particularly noteworthy. This EEG case study not only offers a unique perspective on the physiological effects of LKM but also underscores the potential of wearable technology in gathering extensive data. The “Neurophysiology of the intervention strategies of Awareness Training Program” (Gao, Leung et al.) further complements the exploration of traditional Buddhist practices, emphasizing the impact of such interventions on emotion regulation.

While the majority of the research is centered around meditation and its various forms, the issue also recognizes the importance of broadening its neuroscientific spectrum. Studies like “Inhibitory dysfunction in temporal lobe epilepsy (TLE) patients” (Yu et al.) and “Classification of temporal lobe epilepsy based on neuropsychological tests” (Meng et al.) delve into the realm of epilepsy. By incorporating these, the issue ensures comprehensive coverage, intertwining normative meditation practices with clinical neurological conditions.

Furthermore, the bridging of traditional neuroscience with modern machine learning techniques is evident in the “EEG-based investigation of effects of mindfulness meditation training” (Shang et al.). Indeed, advances in the neuroscience of epilepsy and in machine learning, which is the foundation for AI, can eventually aid in a more evidence-based classification of mindfulness meditation. This classification can also have an impact on the neural measures in clinical psychology practice (Ngan and Cheng). All of this research not only offers fresh insights into the effects of mindfulness meditation but also paves the way for more objective studies.

The potential for large-scale data collection on meditators, especially with the use of wearable devices (Wong et al.), presents both opportunities and challenges. It is believed that AI, like large language models and big data, can provide invaluable insights into which neural indices can best estimate meditation (Ngan and Cheng; Shang et al.). Current cumulative research can pave the way for advanced research methods. Given the reservations of some meditators about their privacy and their occasional hesitance to participate in studies, ethical considerations have to be considered carefully during data gathering and analysis. It is essential to adhere to established ethical guidelines and best practices on personal and sensitive information.

Despite the progress made in this Research Topic, significant challenges remain to be addressed. One of the primary challenges is bridging the gap, especially in terms of theories and terminology, between modern neuroscience and ancient practices, such as those in the Buddhist tradition. The differing theoretical frameworks and foundational concepts often lead to communication barriers. Furthermore, there are methodological challenges in identifying and defining the subjective experiences of advanced meditators and verifying their profound mental states.

Overall, this edition provides a comprehensive perspective and paves the way for upcoming innovations in mental health. It gives an example of the blending of ancient traditions with neuroscientific research techniques, including the use of advanced neuroimaging techniques. This publication signifies a notable advancement in understanding meditation, fostering optimism that as we delve deeper. Based on a more objective methodology, it is estimated that scientists can gain clearer insights into human psychology and spirituality to enhance modern-day mental health practices.

## Author contributions

JG: Writing—original draft. CC: Writing—review & editing. LZ: Writing—review & editing. ET: Writing—review & editing.

